# Effects of Goal Management Training on self-efficacy, self-esteem, and quality of life for persons with schizophrenia spectrum disorders

**DOI:** 10.3389/fpsyg.2024.1320986

**Published:** 2024-03-07

**Authors:** Marie Bjørntvedt Øie, Ingvild Haugen, Jan Stubberud, Merete Glenne Øie

**Affiliations:** ^1^Department of Psychology, University of Oslo, Oslo, Norway; ^2^Research Division, Innlandet Hospital Trust, Brumunddal, Norway; ^3^Department of Research, Lovisenberg Diaconal Hospital, Oslo, Norway

**Keywords:** schizophrenia, psychosis, cognitive remediation, goal management training, executive function, self-efficacy

## Abstract

Persons with schizophrenia often show executive dysfunction assessed with both subjective (self-report) and objective (neuropsychological tests) measures. In a recent randomized controlled trial (RCT), subjective executive functioning in everyday life was improved following Goal Management Training (GMT). The aim of the current study is to investigate the potential of GMT to improve secondary well-being outcomes from that RCT, including self-esteem, self-efficacy, and quality of life in persons with schizophrenia spectrum disorders. Since well-being is frequently lower in persons with schizophrenia compared to healthy individuals, further knowledge about well-being as an outcome after cognitive remediation may have implications for clinical treatment. Sixty-five participants were randomly assigned to GMT (*n* = 31) or a waiting list control condition (*n* = 34). Assessments were conducted at baseline (T1), immediately after the intervention (T2–5 weeks), and at six-month follow-up (T3). Measures included the Rosenberg Self-Esteem Scale, the Perceived Quality of Life Scale, and the General Self-Efficacy Scale. Results were analyzed using a linear mixed model analysis for repeated measures. There were no significant effects of GMT on self-esteem or quality of life. Only the GMT group showed a significant increase in self-efficacy that was most evident at six months follow-up, *F*(1, 34) = 10.71, *p* = 0.002, *d =* 0.71. Improved self-efficacy was found to correlate significantly with a reduction in perceived executive dysfunction in an exploratory *post hoc* analysis. Our findings demonstrate the potential of GMT in improving self-efficacy in schizophrenia

**Clinical trial registration:**https://clinicaltrials.gov, NCT03048695.

## 1 Introduction

Cognitive remediation (CR), is structured behavioral training that aims to improve cognition and real-world function and is recommended with support from high quality evidence in recent international clinical guidelines for the treatment of schizophrenia (Bowie et al., 2020; Vita et al., 2022). CR may take several forms, including techniques thought theoretically to restore cognitive functions emphasizing mass practice of specific cognitive tasks and compensatory techniques such as learning mental strategies to help work around cognitive difficulties (Allott et al., 2020b). Most existing CR interventions for persons with schizophrenia include elements of both restorative and compensatory techniques. For example, in CR based on mass practice of cognitive tasks, the inclusion of an active therapist encouraging, and making explicit, mental strategies has emerged as a prerequisite for effects to be generalized to daily life function (Bowie et al., 2020; Vita et al., 2021). Even though many of today’s CR interventions for persons with schizophrenia use a combination of mass practice and training of both specific and more general strategies, few evaluations have been undertaken of primarily top-down meta-cognitive strategy training as a stand-alone intervention (Medalia and Saperstein, 2013; Vizzotto et al., 2021). However, a review of compensatory cognitive remediation, which included meta-cognitive strategy training, showed improvements in daily life function similar to the research literature on mostly restorative CR approaches (Allott et al., 2020b). CR programs focusing on meta-cognitive strategy training, such as Goal Management Training (GMT) (Robertson, 1996; Levine et al., 2000), are recommended for executive dysfunction following brain injuries (Cicerone et al., 2019; Jeffay et al., 2023). In addition, as effective executive function may be beneficial to the performance of other cognitive tasks, CR for persons with schizophrenia should address executive function (Wykes and Reeder, 2005).

Executive functioning is essential to many aspects of everyday life, and is one of the most frequently impaired cognitive domains in schizophrenia spectrum disorders as measured with neuropsychological tests (objective executive function) (Diamond, 2013; East-Richard et al., 2020). Also, compared to healthy controls, persons with schizophrenia report significantly more executive difficulties in everyday life (subjective executive function) (Bulzacka et al., 2013). Despite methodological challenges with self-report, assessment of subjective executive function is important because it captures experiences of struggling to organize and execute daily activities that may not be evident in examiner-guided and structured test settings (Sbordone, 2014; Friedman and Banich, 2019). In addition, subjective assessment of executive function may perhaps be better suited than objective measures to capture change after meta-cognitive strategy training aimed primarily at improving symptoms of executive dysfunction in real-world situations.

GMT is a meta-cognitive strategy training CR program that targets attentional control and problem-solving and has been shown to improve goal-directed behavior in persons with neurological or mental disorders and executive dysfunction (Stamenova and Levine, 2018). Our group recently conducted an RCT with GMT for individuals with recently diagnosed schizophrenia spectrum disorders or psychosis risk syndromes and found that the intervention improved subjective executive functioning in everyday situations, which was the primary outcome of the trial, and reduced symptoms of anxiety and depression (Haugen et al., 2022). However, the effects of GMT on well-being (e.g., self-esteem, self-efficacy, quality of life) in the context of schizophrenia remain to be investigated. Thus, the present study aims to investigate the effects of GMT on secondary endpoints from the original RCT among the subset of participants who were diagnosed with a schizophrenia spectrum disorder.

Psychological constructs such as quality of life, self-esteem, and self-efficacy are all central aspects of well-being (Ruggeri et al., 2020). Persons with schizophrenia report lower levels of well-being compared to healthy samples (Chino et al., 2009; Gleeson et al., 2021). The concept of quality of life covers satisfaction with physical and psychological health, social relationships and level of independence (Savilla et al., 2008). As a disabling disorder affecting independent living, social contact, and multiple aspects of functioning, it is not surprising that individuals with schizophrenia spectrum disorders experience reduced quality of life compared with healthy samples (Murphy and Murphy, 2006; Wartelsteiner et al., 2016). Knowledge of the determinants of quality of life in schizophrenia is therefore crucial in tailoring effective interventions to improve the lives of the affected (Tolman and Kurtz, 2012). Interestingly, a meta-analysis found that psychiatric symptoms showed only a small relationship to quality of life among persons with schizophrenia (Eack and Newhill, 2007). However, there is an indication of a positive association between executive functioning measured with neuropsychological tests and quality of life in schizophrenia (Fujii et al., 2004; Kurtz and Tolman, 2011).

Low self-esteem is an important treatment target in schizophrenia because of its associations with poorer symptomatic and functional recovery (Holding et al., 2013; Evensen et al., 2017). In addition, low self-esteem is associated with more suicidal ideation and poorer quality of life in this patient group (Ritsner et al., 2003; Wartelsteiner et al., 2016). Not many studies exist on self-esteem and CR, but there is some indication from a case study with a person with schizophrenia that self-esteem increases after GMT as a result of increased confidence in the accomplishments of daily tasks (Rose et al., 2008; Levaux et al., 2012; Seccomandi et al., 2020; Lejeune et al., 2021).

Self-efficacy has been found to be reduced in persons with schizophrenia compared with healthy controls (Chino et al., 2009; Grant and Beck, 2009). The deficits in daily functioning seen in persons with schizophrenia may partially be due to perceptions of not having the abilities necessary to succeed (Ventura et al., 2014; Beck et al., 2018). In some studies, better cognitive function has been associated with better self-efficacy, suggesting that cognitive impairment may contribute to the formation of negative beliefs about task accomplishment (Bryce et al., 2019). Self-efficacy may therefore improve from cognitive enhancing treatments if the individual experiences that the use of problem-solving strategies leads to functional accomplishments post treatment (Allott et al., 2020a). If CR can enhance self-esteem and self-efficacy, it may have an important impact on future treatment and adherence in persons with schizophrenia (Wykes et al., 1999; Ventura et al., 2014). However, very few studies have explored well-being as outcomes of CR (Seccomandi et al., 2020; Lejeune et al., 2021).

GMT addresses executive function in everyday situations and our RCT showed that it was superior to treatment as usual for psychosis in alleviating difficulties with initiating, planning, organizing activities, inhibiting automatic responses, attentional shifts, and self-monitoring among people with schizophrenia spectrum disorders or psychosis risk syndromes (Haugen et al., 2022). Executive functions regulate top-down processes of behavior, emotion, and cognition, making them critically important to nearly all aspects of an individual’s everyday functioning (Diamond, 2013). Executive functions are still developing in late adolescence and early adulthood when first episodes of psychosis are typically diagnosed (Freedman and Brown, 2011; Zelazo, 2020). Executive dysfunction may exacerbate challenges in meeting increased expectations of education, work, independent living, and social situations. Thus, a bidirectional interaction between executive dysfunction and development of psychopathology in schizophrenia has been suggested (Zelazo, 2020; Romer and Pizzagalli, 2021). Failing to meet expectations from family, peers, employers or teachers could cause stress and raise the risk of psychotic symptoms (Freedman and Brown, 2011; Shakoor et al., 2016). As a consequence, interventions aimed at executive function may be particularly important for young adults recently diagnosed with schizophrenia (Carruthers et al., 2019; Melle, 2019).

Though not previously investigated, the core idea of GMT is to improve executive functions, giving participants a sense of increased control, which can improve self-confidence in their abilities (Levine et al., 2011). Although conjectural, this may suggest that GMT may support patients with schizophrenia to gain greater benefit from cognitively demanding interventions. Gutierrez-Rojas et al. (2021) found that people with schizophrenia typically report lower levels of happiness and well-being, and higher levels of perceived stress compared to healthy controls. The study also showed that the relationship between subjective happiness and functioning among patients with schizophrenia is influenced by the level of cognitive impairment. Moreover, another recent study found an association between improved subjective executive function and improved personal recovery after a first episode of psychosis (van Aken et al., 2022). These findings suggest that improving cognitive functioning through rehabilitation programs may benefit patients in terms of recovery outcomes related to subjective happiness and functioning.

Due to limited research concerning self-esteem, self-efficacy, and quality of life as outcome measures of meta-cognitive compensatory interventions for schizophrenia, the hypotheses in the current study are exploratory. Additionally, very few GMT studies have included self-esteem, self-efficacy, or quality of life as outcome measures (Stamenova and Levine, 2018). Nonetheless, based on the potential of GMT to provide participants with strategies for better goal achievement in daily life, it is expected that GMT will improve subjectively rated self-esteem, self-efficacy, and quality of life.

*Hypotheses:* GMT improves self-esteem significantly more than treatment as usual for psychosis.

GMT improves self-efficacy significantly more than treatment as usual for psychosis.

GMT improves quality of life significantly more than treatment as usual for psychosis.

## 2 Materials and methods

The present study reports secondary outcomes from a pre-registered RCT investigating the efficacy of GMT for persons with psychosis or psychosis risk on executive functioning (Registered at clinicaltrials.gov NCT03048695). Primary outcomes of GMT on cognition, daily life function, and clinical symptoms have already been published (Haugen et al., 2022). In the original trial, both persons with a schizophrenia spectrum disorder and persons with a psychosis risk syndrome were included (*n* = 81). However, the present analysis of secondary outcomes of well-being includes only data from 65 individuals with schizophrenia spectrum disorders, to ensure a more homogenous sample.

### 2.1 Participants

Participants were recruited among patients referred for assessment and treatment of psychosis at an early discovery and intervention clinic at Innlandet Hospital in Norway. The sample in this analysis consists of 39 males (60%) and 26 females (40%). The participants had a schizophrenia spectrum disorder according to the criteria in the Diagnostic and Statistical Manual of Mental Disorders, DSM-IV-TR (American Psychiatric Association, 2000). Diagnostic eligibility was determined by a clinical psychologist under supervision from a specialist in psychiatry using the Structured Clinical Interview for the Diagnostic and Statistical Manual of Mental Disorders-IV (DSM-IV) Axis 1 disorders, SCID I and the Positive and Negative Symptoms Scale, PANSS (Kay et al., 1987; First et al., 2005). Additional inclusion criteria were age between 16 and 69 years and treatment for psychosis for less than five years, resulting in a young sample of recently diagnosed individuals aged 16 to 44 years (*M* = 25, *SD* = 6.5, median and mode age 24). The average duration of untreated psychosis in the sample was 241 weeks, median 192 and mode 208. Participants had to have self-reported executive dysfunction in intake interviews or a *T*-score > 55 on the Behavior Rating Inventory of Executive Function- Adult version, BRIEF-A (Roth and Gioia, 2005; Løvstad et al., 2016). Exclusion criteria were severe cognitive problems defined as IQ < 70 estimated with the Vocabulary and Matrix Reasoning subtests from the Wechsler Abbreviated Scale of Intelligence, WASI (Wechsler, 1999), or General Ability Index from Wechsler Adult Intelligence Scale, WAIS-IV (Wechsler, 2008), ongoing alcohol or substance abuse, or premorbid and/or comorbid neurological conditions.

### 2.2 Study design and procedures

**Figure 1:**
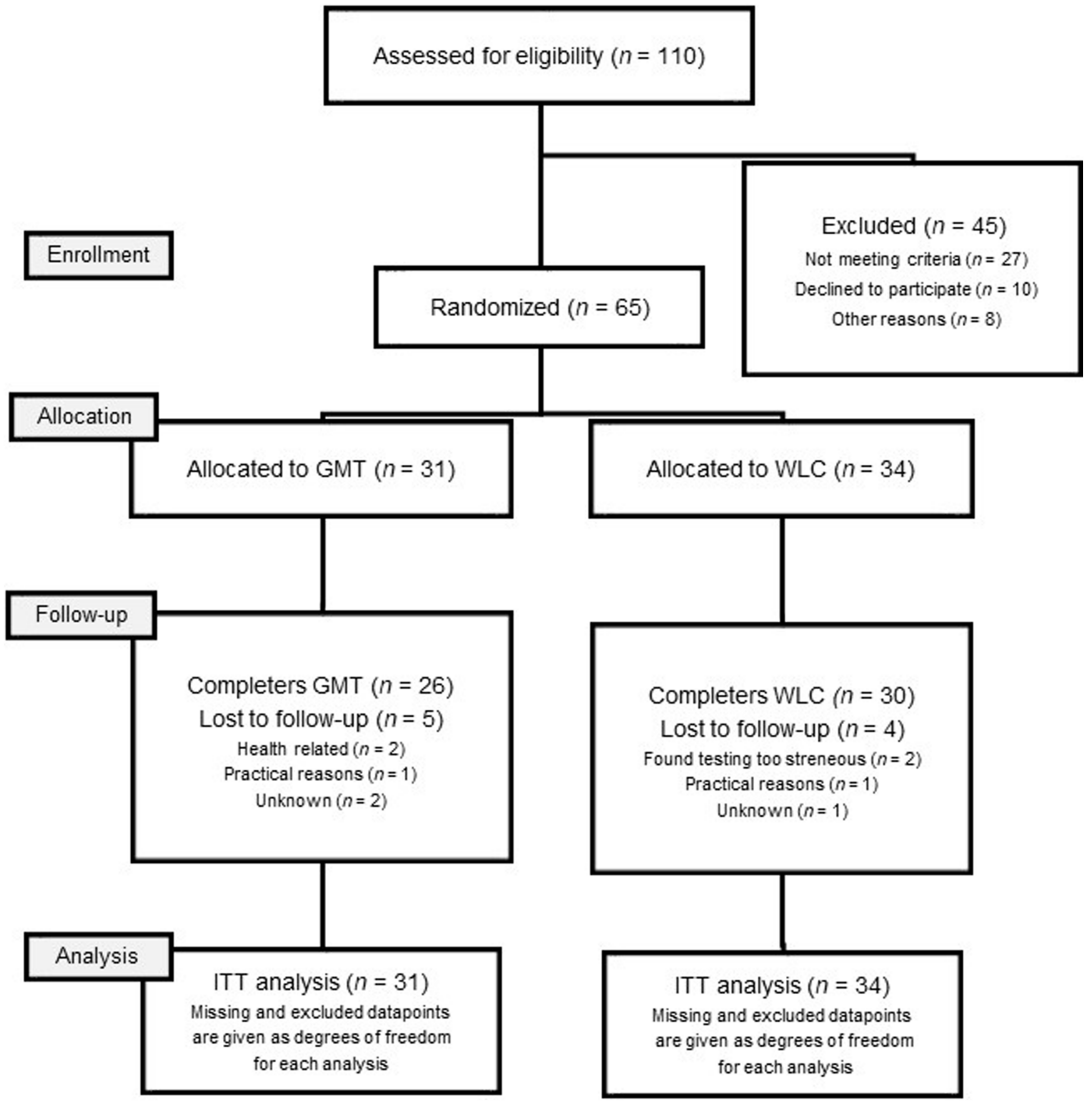
Flowchart of allocation and attrition.

See Figure 1 for a consort flow diagram of the sample according to the guidelines for reporting on parallel group trials (Schulz et al., 2010). All participants completed the baseline assessment (T1) before being randomly assigned to receive GMT in addition to treatment as usual for psychosis (GMT; *n* = 31) or a wait list control group receiving only treatment as usual (WLC; *n* = 34). The WLC group received GMT after the assessments in the study had been completed. Treatment as usual was defined according to national guidelines for psychosis, and often consisted of a combination of medication and psychotherapy (Norwegian Health Authority, 2013). A member of hospital staff not involved in the study assigned participants using computer-generated randomization from https://www.randomizer.org. Reassessment took place immediately after the intervention (T2–5 weeks) and at six months follow-up after the intervention was completed (T3–30 weeks). To ensure blinding, the psychiatric nurses conducting the assessments at T2 and T3 had no access to information regarding treatment conditions.

The study was approved by the Regional Committee for Medical Ethics of South-Eastern Norway (2015/2118) and conducted in accordance with the Helsinki Declaration and Vancouver rules. Informed consent was signed by all participants prior to participation.

### 2.3 The intervention: goal management training

The intervention in the present study was administered according to the Norwegian translation of the GMT protocol (Stubberud et al., 2013). GMT teaches meta-cognitive strategies for attentional control and problem-solving (Levine et al., 2011). The intervention has historical roots in observations of goal neglect in patients with pre-frontal brain injuries who could state their goals, but did not undertake actions necessary to reach those goals (Robertson, 1996; Levine et al., 2011). The theoretical basis of GMT is the assumption that a disruption of sustained attention will lead to distracted, rather than goal driven, behavior – reliant on external cues or habits to perform actions. GMT aims to increase goal driven behavior through learning a compensatory mental strategy in steps such as STOP-FOCUS-STATE GOAL-CHECK. GMT incorporates exercises in attentional control and regulation of arousal, as well as meta-cognitive approaches to problem solving such as dividing up overwhelming tasks and prioritizing goals (Kabat-Zinn, 1990; Levine et al., 2011).

In the present study, GMT was administered in groups of three to eight participants, with nine sessions of 2 h duration each. Sessions were held twice a week by a clinical psychologist together with trained co-therapists with different professional backgrounds including occupational therapists, psychologists, and physicians. GMT sessions consisted of psychoeducation, narrative examples, practical exercises, and mindfulness (Kabat-Zinn, 1990; Levine et al., 2011). Participants were encouraged to develop one individual long-term goal using goal attainment scaling (Ashford and Turner-Stokes, 2006; Krasny-Pacini et al., 2014). Between sessions four and nine, daily text messages with the word STOP reminded participants to rehearse the GMT strategy (Manly et al., 2002; Fish et al., 2007). Aids were PowerPoint slides, participant workbooks, and group discussions. Homework assignments were completed between sessions. Participants who were unable to attend sessions were offered individual catch-up sessions. See Haugen et al. (2022) for theoretical background and a more detailed description of the content of GMT.

### 2.4 Measures

The following measures were used to capture the participants’ subjective experience of self-esteem, self-efficacy, quality of life, and difficulties with executive function in daily life. All questionnaires have been translated into Norwegian in authorized versions.

#### 2.4.1 Self-esteem

Self-esteem was assessed using the Rosenberg Self-Esteem Scale (RSES), a 10-item questionnaire with scores along a 4-point Likert scale (Rosenberg, 1965). The RSES is the most frequently used instrument to measure global self-esteem through self-report, and it is validated in multiple populations, including patients with schizophrenia (Schmitt and Allik, 2005; Lecomte et al., 2006). Good psychometric properties of the scale have been confirmed (Sinclair et al., 2010). Higher scores correspond to higher self-esteem. Negatively worded items are reverse-scored. In the current study, the Cronbach’s alpha coefficient for the scale was α 0.92 at baseline, indicating good internal consistency.

#### 2.4.2 Self-efficacy

Self-efficacy was assessed by the questionnaire General Self-Efficacy Scale (GSES) (Schwarzer and Jerusalem, 1995), which consists of 10 items scored along a 4-point Likert scale. According to Schwarzer (1999), general perceived self-efficacy pertains to optimistic beliefs about being able to cope with different stressors, and the scale measures self-efficacy as a unitary construct. A typical item is “*I can solve most problems if I invest the necessary effort*.” Higher score indicates higher level of self-efficacy. High reliability and construct validity of the scale have been confirmed in various samples and cultures (Leganger et al., 2000; Scholz et al., 2002). In the current study, the scale had a Cronbach’s alpha coefficient of α.85 at baseline, showing good internal consistency.

#### 2.4.3 Quality of life

The Perceived Quality of Life Scale (PQoL) (Patrick et al., 2000) was used as a measure of quality of life in the current study. This scale was generated to measure satisfaction with life among individuals with disabilities and chronic health conditions (Patrick et al., 2000). Nineteen items assess satisfaction with physical, psychological, and social functioning, and an additional 20th item addresses overall happiness (“*How happy are you?*”). The PQoL is an 11-point Likert Scale ranging from 0 (*extremely dissatisfied*) to 10 (*extremely satisfied*). The average score of item 1–19 was highly correlated with item 20, *r* = 1.00 (0.998), indicating good convergent validity. The PQoL also had good internal consistency at baseline in the present sample with a Cronbach’s alpha of α.90.

#### 2.4.4 Subjective executive functioning

The Behavior Rating Inventory for Executive Function – Adult version (BRIEF-A) (Roth and Gioia, 2005) was used in a *post hoc* analysis exploring the association between any significant treatment effects of GMT on well-being and the primary outcome of the original RCT. The BRIEF-A is a 75-item questionnaire measuring difficulties with executive function in everyday situations. A higher score indicates more difficulties. The instrument has shown good test–retest reliability ranging from *r* 0.82–0.93 across nine subscales covering initiation, planning/organizing, inhibition, mental flexibility, working memory, self- and task- monitoring, emotional control, and organization of materials (Roth and Gioia, 2005). The scale showed good internal consistency in the present study at baseline with a Cronbach’s Alpha score of α 0.95 for the total score.

#### 2.4.5 Social functioning scale

Social Functioning Scale, SFS, is a self-reported questionnaire developed for people with schizophrenia (Birchwood et al., 1990). The Norwegian translation of the scale has been shown reliable and valid among people with schizophrenia (Hellvin et al., 2010). The scale consists of seven subscales. Out of these, the two subscales Independence Competency and Independence Performance were used as functional measures in the *post hoc* analysis in the present paper, as these subscales measure 13 activities of daily living. Independence Performance asks how often participants cook food, shop, clean the house, and other central activities of daily living. Independence Competency asks how capable the participants perceive themselves to be in doing the same activities, for example whether they require assistance or not. The subscales Withdrawal, Interpersonal Behavior, Pro-Social Activities and Recreation are thought to cover aspects of social functioning more likely associated with social cognition than executive function (Horan et al., 2011). The final subscale is Employment, which was considered unlikely that GMT could change in six months, since occupational status also depends on external factors. The internal consistency of the Independence Performance subscale, α = 0.83, and of the Independence Competency subscale, α = 0.69, were adequate in the present study.

### 2.5 Statistical analyses

Data were analyzed using the IBM Statistical Package for Social Sciences (SPSS) version 27. The Mann–Whitney U Test for continuous variables and Pearson Chi-Square Test for categorical variables were used for baseline comparisons. A linear mixed model analysis for repeated measures was used to investigate the effects of GMT on the outcome measures: total raw scores on the Rosenberg Self-Esteem Scale (RSES), Perceived Quality of Life Scale (PQoL) and General Self-Efficacy Scale (GSES). Scores on the outcome variables were normally distributed. All partial data were analyzed according to the principle of intention-to-treat (ITT) to yield a statistically robust estimate of the efficacy of the intervention (McCoy, 2017; Schielzeth et al., 2020). The time variable was coded 0 for baseline, 1 for post intervention assessment, and 2 for follow-up assessment, as a linear function was expected for theoretical reasons. A first-order autoregressive covariance matrix was chosen for the repeated measurements. Time, group, and group-by-time interactions were specified as fixed effects. Effects with *p*-values smaller than 0.05 were considered statistically significant. Random subject intercepts were allowed for in the models. Restricted maximum likelihood was used as a method of estimation due to a small sample size and the use of repeated measures. The effect size, Cohen’s *d,* was calculated from the difference between the treatment (GMT) and the control (WLC) group in mean change score from baseline (T1) to six-month follow-up (T3) (Cohen, 1988, 1992). Additional linear mixed models were run to control for any effects of age on changes in well-being as a result of GMT.

Due to the study’s novelty, *post hoc* analyses were performed to explore the association between the change of the measure that showed a significant treatment effect, GSES, and the primary outcome measure in the trial which was executive functioning in daily life (BRIEF-A). In a linear regression analysis, standardized residuals were calculated using baseline (T1) scores as a predictor for follow-up (T3) scores on BRIEF-A and GSES. These standardized residuals were then correlated using a Pearson correlation coefficient.

Similarly, we investigated the relationship between change in self-efficacy and two measures of real-world functioning, namely self-reported Independence Performance and Independence Competency in activities of daily life from the Social Functioning Scale (Birchwood et al., 1990), which showed a larger effect after GMT compared to treatment as usual in the *post hoc* analysis from the main RCT (Haugen et al., 2022).

## 3 Results

### 3.1 Baseline characteristics

At baseline, the levels of self-esteem in the sample were *M* = 2.31, *SD* = 0.09, and levels of quality of life were *M* = 4.94, *SD* = 0.19. Levels of self-efficacy were *M* = 2.55, *SD* = 0.06.

#### 3.1.1 Comparison of treatment groups at baseline

At baseline, the GMT participants reported higher mean self-esteem, *M* = 2.58, *SD* = 0.85, compared to the WLC participants, *M* = 2.15, *SD* = 0.57, *U* = 557.00, *z* = 2.21, *p* = 0.027, *r* = 0.29. The groups did not differ in quality of life or self-efficacy. The GMT group reported more executive complaints at baseline, whereas the WLC group experienced a higher level of negative symptoms. See Table 1 for details of the baseline characteristics. The groups were otherwise comparable in demographical, clinical, and cognitive variables. See Table 1 for details.

**Table 1:** Baseline characteristics of the sample (*n* = 65).

	GMT (*n* = 31) *M (SD)*	WLC (*n* = 34) *M (SD)*	Sig.
Age (years)	26 (6.68)	25 (6.47)	0.580
Sex (*n*/%)			0.102
Female Male	15/48.4% 16/51.6%	11/32.4% 23/67.6%	
Education (years)	12.81 (1.96)	12.91 (1.69)	0.629
Estimated IQ	98.48 (15.46)	97.61 (12.67)	0.917
Executive complaints: BRIEF-A Total *T*-score	70.22 (9.43)	64.36 (11.38)	**0.031**
Diagnosis (n/%)			0.512
Schizophrenia	12/38.7%	17/50.0%	
Schizoaffective disorder	6/19.4%	8/23.5%	
Schizophreniform episode	4/12.9%	2/5.9%	
Delusional disorder	0/00.0%	1/2.9%	
Psychosis NOS	9/29.0%	6/17.6%	
PANSS			
Positive symptoms	3.11 (0.87)	2.88 (0.81)	0.300
Negative symptoms	2.36 (0.76)	2.76 (0.83)	**0.044**
Depressive symptoms	3.47 (1.01)	3.45 (0.90)	0.776
Disorganized symptoms	2.35 (0.58)	2.41 (0.66)	0.754
Excited symptoms	2.28 (0.61)	2.04 (80.65)	0.101
Duration of untreated psychosis (weeks)	253.81 (278.84)	229.68 (210.95)	0.927
Hospitalizations (*n*)	3.92 (4.60)	3.16 (5.63)	0.702
Months in hospital	5.45 (9.46)	5.91 (6.88)	0.504
Antipsychotic medication (n/%)	22/71.0%	23/67.6%	0.772

Bold signifies statistical significance.

#### 3.1.2 Completers versus non-completers

Attrition from baseline to six months follow-up was 13.8%, leaving 56 participants (86.2%) who completed all assessments. Completers and non-completers were comparable on clinical measures, except non-completers had higher baseline scores than completers for some of the symptom categories measured with the PANSS. The differences were statistically different for mean negative symptoms at baseline (completers *M* = 2.46 vs. non-completers *M* = 3.24, *U* = 122.00, *z* = −2.48, *p* = 0.013, *r* = −0.31), depressive symptoms (completers *M* = 3.36 vs. non-completers *M* = 4.07, *U* = 148.00, *z* = −1.99, *p* = 0.047, *r* = −0.25) and excited symptoms (completers *M* = 2.09 vs. non-completers *M* = 2.56, *U* = 137.00, *z* = −2.20, *p* = 0.028, *r* = −0.27). Further, we found no significant differences at baseline in the outcome variables self-esteem (completers *M* = 22.65 total score vs. non-completers *M* = 27.22, *U* = 140.00, *z* = −1.73, *p* = 0.083, *r* = −0.23) or quality of life (completers *M* = 94.40 total score vs. non-completers *M* = 105.25, *U* = 145.50, *z* = −1.02, *p* = 0.317, *r* = −0.14). However, there was a significant difference in total score for self-efficacy at baseline (completers *M* = 24.79 total score vs. non-completers *M* = 27.89, *U* = 119.00, *z* = −2.07, *p* = 0.038, *r* = −0.28). As a result, linear mixed models analyses were performed using the scores of completers only, showing similar results for the group x time interaction as the ITT analysis for self-efficacy *F*(1, 32) = 9.43, *p* = 0.004, self-esteem *F*(1, 37) = 0.32, *p* = 0.575 and quality of life *F*(1,35) = 0.82, *p* = 0.372.

#### 3.1.3 Treatment effects

**Figure 2:**
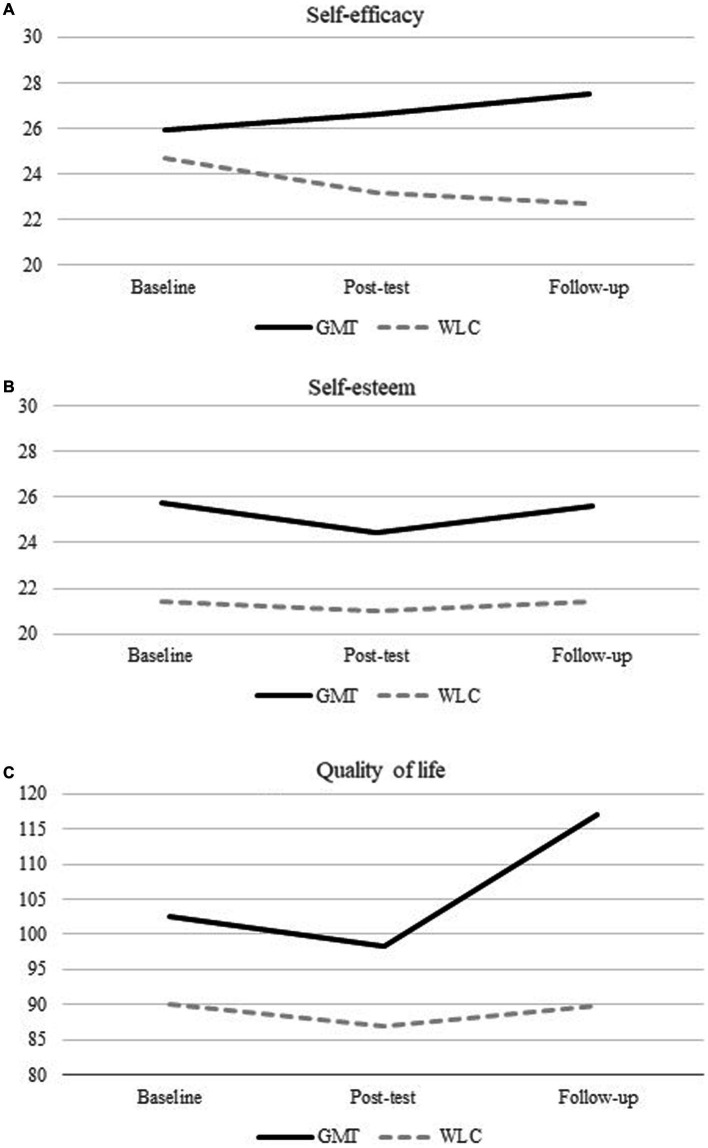
Results from the linear mixed model analysis.

A significant increase in total self-efficacy across time was found in the GMT group only, *F*(1, 34) = 10.71, *p* = 0.002, *d =* 0.71. This was considered a medium effect size (Cohen, 1988). There were no significant treatment group × time interactions for total self-esteem, *F*(1, 39) = 0.25, *p* = 0.621, *d* = 0.03, or total quality of life, *F*(1, 40) = 1.42, *p* = 0.241, *d* = 0.49. Results from the linear mixed models analysis are presented in Table 2 and Figure 2.

**Table 2:** Linear mixed model analysis of repeated measures of self-esteem, self-efficacy, and quality of life.

	GMT (*n* = 31) Mean scores			WLC (*n* = 34) Mean scores			Group × Time interaction					
	Baseline	Post test	Follow-up	Baseline	Post test	Follow-up	Df	*b* (GMT)	SE	95% CI	Sig.	*d*
Self-efficacy	25.96	26.63	27.53	24.70	23.17	22.72^a^	33.95	2.30	0.70	0.87, 3.73	**0.002**	0.71
Self-esteem	25.77	24.43	25.59	21.41	21.04	21.44	38.68	0.44	0.87	−1.33, 2.20	0.621	0.03
Quality of life	102.50	98.41	116.93	90.14	86.96	89.81	39.50	6.06	5.10	−4.24, 16.37	0.241	0.49

Higher raw scores indicate better outcomes. GMT = Goal Management Training, WLC = Wait list control group.

^a^Significant main effect of time.

There was also a significant main effect of time found for self-efficacy *F*(1, 34) = 4.83, *p =* 0.035, due to a small decline in self-efficacy in the WLC group over the course of the study. There was no main effect of group. Since the GMT group and the WLC group differed in negative symptoms and self-esteem at baseline, these variables were added as covariates to check for any influence. The effect of GMT on self-efficacy did not change when controlling for negative symptoms and self-esteem. There was a main effect of age on self-efficacy nearing significance, *F*(1, 59) = 3.93, *p* = 0.052. However, there was no significant interaction effect between treatment group x time x age, *F*(1, 34) = 0.19, *p* = 0.666. There was no main effect of age on self-esteem, *F*(1, 60) = 1.88, *p* = 0.176, nor any significant interaction effects of group × time × age, *F*(1, 37) = 0.05, *p* = 0.833. There was no main effect of age on quality of life *F*(1, 58) = 0.78, *p* = 0.382, nor any significant interaction effect of group × time × age *F*(1, 41) = 0.00, *p* = 0.963.

#### 3.1.4 *Post hoc* exploratory analyses

*Post hoc* analyses revealed a strong significant negative correlation, *r* (df 59) = −0.72, *p* = 0.001, bias corrected 95% CI: −0.87, −0.43, between total GSES and total BRIEF-A standardized residuals after linear regression analyses of baseline to follow-up scores. For the GMT group the correlation was *r* = −0.79 compared to *r* = −0.39 in the WLC group. Hence, improved self-efficacy was found to correlate significantly with a reduction in perceived executive dysfunction. Change in self-efficacy was not correlated with change in regularity *r (df 33)* = 0.22, *p* = 0.218, or ability to perform activities of independent living *r (df 33)* = 0.24, *p* = 0.182 measured with the self-reported Social Functioning Scale.

## 4 Discussion

The present RCT is the first to investigate the effect of the meta-cognitive strategy training GMT on self-esteem, self-efficacy, and quality of life in persons with schizophrenia spectrum disorders. At baseline, the sample showed average levels of self-esteem similar to normative samples (Sinclair et al., 2010). However, levels of quality of life and self-efficacy were lower than in healthy samples (Patrick et al., 2000; Scholz et al., 2002). Participation in GMT led to a significant increase in perceived self-efficacy immediately after the intervention which increased in the 6 months following the intervention. Enhanced self-esteem and quality of life after GMT were expected, but despite improvement in the intervention group, there was no significant effect compared to the WLC group.

The finding of increased self-efficacy after GMT is logical given the potential of GMT to offer strategies to master daily tasks (Levaux et al., 2012; Stamenova and Levine, 2018; Vizzotto et al., 2021). The finding suggests that participating in GMT may have important implications for how individuals perceive their ability to function in daily life. According to the theory of Bandura (1977) self-efficacy is induced and altered through mastery experiences, vicarious experiences, verbal persuasion, and emotional and physiological states. It is possible that GMT contributed to increased self-efficacy through these mechanisms. For example, feedback from the participants after the intervention indicated that GMT led to valuable mastery experiences in daily life. One participant told the group “I came home from the shop with everything I planned to buy for the first time in my life!” Moreover, the GMT therapists and the other group members may have provided verbal persuasion and vicarious experiences of mastery through the sharing of experiences (Bandura, 1977; Cella et al., 2014; Bowie et al., 2020; Lejeune et al., 2021). A sense of normalization, belonging, and a reduced feeling of isolation is reported to be valuable aspects of group interventions delivered to patients with schizophrenia (McCay et al., 2006; Contreras et al., 2016). Finally, the repetitive performance of the strategy and mindfulness exercises in GMT may have contributed to extinguishing fear arousal and emotional reactions associated with facing novel tasks (Bandura, 1977, 1986; Levine et al., 2011).

The *post hoc* analysis revealed significant correlations between perceived self-efficacy and subjective executive functioning, indicating that GMT-related changes in executive functioning are related to changes in self-efficacy. This may indicate that self-efficacy is a mediator of improved executive function after GMT. However, caution in the interpretation of results is warranted due to the exploratory nature of these *post hoc* analyses. For example, conclusions related to directionality are not possible. There is some indication that defeatist beliefs similar to low self-efficacy may mediate the relationship between cognition, negative symptoms of psychosis, and real-world function (Grant and Beck, 2009). There are also some CR studies using drill and strategy approaches that have used self-efficacy as an outcome measure, but at present not enough data exists to conclude what role self-efficacy may play in CR for schizophrenia (Bryce et al., 2018; Lejeune et al., 2021). Increasing knowledge of the mechanisms in both compensatory and restorative CR is important for improving interventions to reach their full potential and deciding on the most successful treatment (Cella and Wykes, 2019). The *post hoc* analysis revealed no significant relationship between improvement in self-efficacy and change in self-reported activities of independent living. As such, self-efficacy does not seem to be a driver of change after GMT for functional measures beyond that of subjective executive functioning. As there was improvement in functional measures in the main trial in both the group of participants receiving GMT and the group receiving only treatment as usual, it may be that the findings of improved subjective executive functioning and self-efficacy are coincidental. However, the effect sizes are robust and the findings are in line with theoretical assumptions behind the mechanisms in GMT (Haugen et al., 2022). Furthermore, methodological challenges with the small sample size and distal functional measures not being sensitive enough to detect change may prevent conclusions about the generalization of GMT to real-world function.

Contrary to our hypotheses, no significant treatment effects emerged for self-esteem or quality of life. One explanation might be that self-esteem and quality of life are more global and multifaceted constructs than self-efficacy (Bailey, 2003; Haraldstad et al., 2019). Quality of life encompasses subjective feelings of satisfaction with life as well as objective indices and resources (Eack et al., 2007). It covers physical and psychological health, social relationships, and the level of independence (Savilla et al., 2008; Hasan and Tumah, 2019). It is therefore likely to be more strongly influenced by circumstances in the participants’ lives not targeted by GMT. Similarly, the Rosenberg Self-Esteem Scale (RSES) used in the present study is designed to measure global self-esteem, reflecting a general subjective sense of self consisting of multiple domains that may or may not be influenced by GMT (Rosenberg et al., 1995). Since GMT focuses on the accomplishment of daily tasks, self-efficacy may be a more proximal measure than self-esteem. In addition, self-esteem may be harmed by social stigma in other ways compared to self-efficacy in individuals with schizophrenia (Jahn et al., 2020). There is some indication that self-esteem is more closely related to affective processes, whereas self-efficacy is more closely related to motivational processes (Chen et al., 2004). Furthermore, the sample in the current study reported self-esteem scores comparable to normative samples initially, leaving less room for improvement whereas levels of self-efficacy were reduced compared to healthy samples (Patrick et al., 2000; Scholz et al., 2002; Sinclair et al., 2010).

Because self-efficacy is based on appraisals of past success and failure, it can be argued to be more dynamic in nature and change more quickly than the other two measures (Bandura, 1977). In fact, self-esteem shows little improvement after treatment of schizophrenia spectrum disorders across studies (Lecomte et al., 2006; Gleeson et al., 2021). Although self-esteem and quality of life are assumed to be responsive to treatment, the time until follow-up measurements in the present study may have been too short to detect or experience change (Robson, 1988). It is possible that if the participants in GMT master more daily life tasks, they may get more positive attention and feedback from others, which in the long run may improve self-esteem and quality of life. High self-efficacy may enhance motivation and participation in positive behaviors important for self-esteem and quality of life (Zahodne et al., 2015). It may also aid in the acquisition and maintenance of functional and social roles (Suzuki et al., 2011). Thus, self-efficacy may result in improved self-esteem and quality of life in the longer term (Hansson, 2006; Ritsner et al., 2012).

### 4.1 Implications

Some individuals with schizophrenia lack confidence in their ability to succeed in daily life because they have experienced repeated failure in the past, due in part to cognitive difficulties (Medalia and Richardson, 2005). Thus, the finding in the present study that a five-week, group-based GMT intervention led to lasting improvement in self-efficacy in patients with schizophrenia spectrum disorders, has promising implications for meta-cognitive strategy training in this patient group. Especially considering if improved self-efficacy proves to be beneficial for overall psychiatric treatment and adherence in persons with schizophrenia (Wykes et al., 1999; Ventura et al., 2014). The positive implications for GMT are further supported by a recent study showing improvements in subjective executive function were associated with personal recovery after first episode of psychosis (van Aken et al., 2022). However, further knowledge is needed about the role of defeatist beliefs, self-efficacy, and self-esteem as potential moderators of CR (Seccomandi et al., 2020; Gutierrez-Rojas et al., 2021).

Nonetheless, GMT did not improve self-esteem and quality of life in this patient group implying that the intervention can benefit from further development perhaps incorporating elements from other interventions specifically addressing well-being. For example, CR taking the participants cognitive strengths, rather than deficits, as a starting point has been recommended (Allott et al., 2020a). Although GMT normalizes executive challenges and provides opportunities for celebrating achievement in group sessions, some may still experience negative effects on well-being. In the future, qualitative investigations of GMT may shed light on both positive and potentially harmful effects of the intervention on well-being (Rose et al., 2008; Nordby et al., 2021). Additionally, it has been suggested that to foster self-efficacy CR should ensure achievement of gradually more difficult tasks and some participants may need more individual follow-up during GMT if they are to achieve successful application of the meta-cognitive strategy in increasingly challenging situations (Bryce et al., 2018, 2019).

The integration of GMT into comprehensive treatment and educational or vocational interventions may improve generalization to real-world function, and perhaps as a result well-being, as not all participants have the same relevant opportunities for practicing what they learn in GMT (Holshausen et al., 2014; Bowie et al., 2020). In the present study, GMT was offered as an add-on to treatment as usual and goals set by participant for GMT were not integrated into overall treatment (Haugen et al., 2022). Perhaps integrating meta-cognitive strategy training better in early intervention services and combining it with other psychosocial interventions will increase effects on well-being, taking particular care to ensure agreement around overarching goals of treatment (van Duin et al., 2019, 2021; Solmi et al., 2023).

### 4.2 Strengths and limitations

The randomized design with masking of conditions and a six-month follow-up assessment are considered the main strengths of this study. The small sample size is a limitation of the study. Missing data from the outcome variables is an additional limitation of the study as it may raise a concern about bias. However, there were few systematic differences between the participants who completed all questionnaires and those who did not which reduces the likelihood of bias. In addition, mixed model analysis can accommodate missing data points and provide unbiased estimates under the assumption of missing at random (Schielzeth et al., 2020). The present study is based solely on subjective ratings to capture how self-esteem, self-efficacy, and quality of life were experienced by the individuals. However, the validity and accuracy of self-report measurements in schizophrenia research may be affected by mood disturbances and reduced self-awareness (Harvey and Pinkham, 2015).

## 5 Conclusion

Five weeks of GMT delivered in small groups to persons with schizophrenia spectrum disorders led to improvements in self-efficacy that lasted at least six months after intervention. No significant effects of GMT were found on self-esteem or quality of life.

## Data availability statement

The raw data supporting the conclusions of this article will be made available by the authors, without undue reservation.

## Ethics statement

The studies involving humans were approved by Regional Committee for Medical Ethics of South-Eastern Norway (2015/2118). The studies were conducted in accordance with the local legislation and institutional requirements. The participants provided their written informed consent to participate in this study.

## Author contributions

MBØ: Formal analysis, Writing – original draft. IH: Formal analysis, Investigation, Writing – review & editing. JS: Conceptualization, Methodology, Writing – review & editing. MGØ: Conceptualization, Funding acquisition, Project administration, Supervision, Writing – review & editing.
